# Stage‐dependent conditional survival and failure hazard of non‐metastatic nasopharyngeal carcinoma after intensity‐modulated radiation therapy: Clinical implications for treatment strategies and surveillance

**DOI:** 10.1002/cam4.3917

**Published:** 2021-05-06

**Authors:** Jingbo Wang, Xiaodong Huang, Shiran Sun, Kai Wang, Yuan Qu, Xuesong Chen, Runye Wu, Ye Zhang, Qingfeng Liu, Jianghu Zhang, Jingwei Luo, Jianping Xiao, Li Gao, Guozhen Xu, Chen Hu, Ye‐Xiong Li, Junlin Yi

**Affiliations:** ^1^ Department of Radiation Oncology National Cancer Center/National Clinical Research Center for Cancer/Cancer Hospital, Chinese Academy of Medical Sciences and Peking Union Medical College Beijing China; ^2^ Division of Biostatistics and Bioinformatics Sidney Kimmel Comprehensive Cancer Center, Johns Hopkins University School of Medicine Baltimore MD USA

**Keywords:** conditional survival, hazard analysis, intensity‐modulated radiotherapy, nasopharyngeal neoplasms, survival analyses

## Abstract

**Purpose:**

Conditional survival (CS) and failure hazard estimations can provide important dynamic prognostic information for clinical decision‐making and surveillance counseling. The current study aimed to investigate the CS and dynamic failure hazard in non‐metastatic nasopharyngeal carcinoma (NPC) treated with intensity‐modulated radiotherapy (IMRT).

**Methods:**

Conditional overall survival (COS) and progression‐free survival (CPFS) estimates adjusted for age and gender against each AJCC 8th stage were calculated. Multivariable Cox regression (MCR) models were fitted in the entire population at baseline and subsequently separate MCR models were fitted in patients who have maintained event‐free time of 1 to 10 years to generate respective hazard ratio (HR). Annual hazard rates of death and progression over 10 years for each stage were also estimated.

**Results:**

A total of 1993 patients were eligible for analysis. The estimated 5‐year OS and PFS for entire cohort were 79.0% and 70.7% at initial diagnosis. After 5 years of event‐free follow‐up, additional 5‐year COS and CPFS increased to 85.9% and 85.5%, respectively. Stage I/II maintained dramatically favorable CS and low hazard (< 5%) of death and progression over time. Relative to stage I/II, stage III manifested non‐significantly higher failure hazard for the first 3 years of survivorship and approached to similar level of stage I/II afterwards. Stage IVA presented most impressive improvement in terms of both COS (∆=9.8%) and CPFS (∆ = 16.8%) whereas still drastically inferior to that of stage I‐III across all conditional time points. After 4 years of follow‐up, progression hazard of stage IVA became relatively steady of approximate 6%.

**Conclusions:**

Survival prospect of non‐metastatic NPC improves over years with distinct dynamic patterns across stages, providing important implications for personalized decision‐making in terms of both clinical management and surveillance counseling. Stage‐dependent and hazard‐adapted clinical management and surveillance are warranted.

## Graphical abstract

This study aimed to investigate the conditional survival (CS) and dynamic failure hazard in non‐metastatic nasopharyngeal carcinoma (NPC) treated with intensity‐modulated radiotherapy (IMRT). Survival prospect of non‐metastatic NPC improved over years with distinct dynamic patterns across stage, providing important implications for personalized decision‐making in terms of both clinical management and surveillance counseling. Stage‐dependent and hazard‐adapted clinical management and surveillance are warranted.

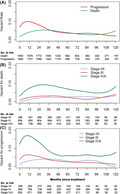

## 1 INTRODUCTION

Nasopharyngeal carcinoma (NPC) is the most prevalent head and neck cancer in China, presenting distinct biological behavior and clinical outcome compared with other entities in head and neck.^1^ Intensity‐modulated radiotherapy (IMRT) has been widely accepted as the mainstay radiotherapy technique in NPC, leading to significantly improved tumor control as well as reduced toxicity in comparison to other conventional radiotherapy techniques.^2^, ^3^, ^4^, ^5^ In the era of IMRT based multidisciplinary management, the 5‐year overall survival (OS) of non‐metastatic NPC population has increased to approximately 80% as traditionally estimated at initial diagnosis.^6^, ^7^, ^8^ However, since the failure hazard during post‐therapy follow‐up is not a constant and the survival probability of cancer patient evolves over time, static survival estimates only at initial diagnosis no longer fulfills clinical demands on the condition of long‐term survivorship.

Conditional survival (CS) is an alternative and dynamic estimate of survival, representing the probability that a patient survives certain additional years beyond a predefined time interval.^9^ Annual failure hazard is a measure that dynamically illustrates the absolute hazard of event during the follow‐up period. On the basis of CS and dynamic failure hazard estimations, care providers are able to obtain a dynamic and more accurate outlook of survival for patients.^10^, ^11^ These dynamic estimates provide paramount prognostic implications to guide individualized clinical management such as determining the indication and intensity for consolidation therapy after IMRT. Moreover, these dynamic prognosis data assist the hazard‐adapted surveillance schedule‐making to lessen patients’ anxiety on disease and further to decrease unnecessary financial cost.

Currently, CS has been investigated in multiple types of cancer, such as lung, breast, gastric, melanoma, glioblastoma, renal cell, head/neck and so on.^9^, ^10^, ^11^, ^12^, ^13^, ^14^, ^15^, ^16^, ^17^, ^18^, ^19^ However, conditional survival and dynamic failure hazard of NPC in IMRT era and the context of 8^th^ AJCC stage is still poorly understood, impeding the access to individualized clinical practice such as post‐IMRT consolidation therapy and surveillance recommendation after initial treatment.

Therefore, it is imperative to investigate the dynamic prognosis on the basis of conditional survivorship and failure hazard for NPC in the era of IMRT and AJCC 8th TNM stage. These data will provide critical prognostic information to optimize clinical decision‐making and guide surveillance counseling for non‐metastatic NPC.

## 2 PATIENTS AND METHODS

### 2.1 Study population and data extraction

Consecutive patients with NPC receiving IMRT in our institution between 2003 and 2017 were selected for analysis. Patient, disease, treatment and follow‐up data were extracted from the in‐house database. Patients with distant metastases or secondary primary tumor in other sites at diagnosis were excluded. Clinical records and radiological images of patients were reviewed to re‐stage their diseases according to AJCC 8th edition criteria. This study was approved by the local institutional review board (IRB).

### 2.2 Statistical analysis

Overall survival (OS) was defined as the time elapsing from start of treatment to the latest follow‐up or death. Progression‐free survival (PFS) was defined as the duration from start of treatment to the date of first progression or death. Conditional overall survival (COS) represents the probability of surviving further *t* years, given that this patient has already survived s years,^10^ which is calculated as:

$$\text{COS}\left(t|s\right)=\frac{S\left(s+t\right)}{S\left(s\right)}.$$

Similarly, conditional progression‐free survival (CPFS) was defined as the likelihood of survival without progression in additional *t* years given that this patient has survived *s* years without progression.

Multivariable Cox regression (MCR) models were fitted in the entire population at baseline and subsequently separate MCR models were fitted in patients who have maintained event‐free time of 1 to 10 years.^14^ The variables of interest included stage, age and gender. Survival estimates adjusted for age and gender against each stage were calculated and plotted by using the corrected group prognosis method.^9^, ^20^, ^21^ To further access the relative hazard ratios (HR) of patients with more advanced stage against early stage with regard to death and progression, all of three variables of interest were included as explanatory variables in respective MCR models fitted in relevant patient settings. Schoenfeld residuals test was adopted to examine the validity of the proportional hazard (PH) assumption. Annual hazard was estimated as the number of events in certain year divided by accumulated follow‐up time of all patients at risk in that year. Smoothed annual hazard curves were plotted by applying kernel‐based methods.^22^

Statistical analyses were conducted with SPSS 22.0 (IBM Inc.), Graphpad Prism 6.0C (GraphPad Software, Inc.) and R 3.6.2 (R Foundation for Statistical Computing). All tests of statistical significance were two sided.

## 3 RESULTS

### 3.1 Patient characteristics and survival estimates at baseline

A total of 1993 patients who received IMRT and had complete data were included for analysis. General characteristics of the study cohort are shown in Table 1. In view of the relative small number of patients with stage I disease and non‐significantly different outcome between stage I and stage II reported by sizable studies,^6^, ^8^ we combined stage I and stage II patients to generate a subgroup of stage I/II for the following analyses. With the median follow‐up time of 60 months, the 5‐year OS and PFS estimated at baseline was 79.0% (95% confidence interval, CI: 77.0% to 80.9%) and 70.7% (95% CI: 68.5% to 72.8%), respectively.

**TABLE 1:** General characteristics of the study population

Characteristics	Number (%)
Age	
Median (range)	47 (4, 83)
≦18	78 (3.9%)
19–40	528 (26.5%)
41–46	1,095 (54.9%)
> 60	292 (14.7%)
Gender	
Male	1,464 (73.5%)
Female	529 (26.5%)
T stage	
T1	307 (15.4%)
T2	340 (17.1%)
T3	782 (39.2%)
T4	564 (28.3%)
N stage	
N0	175 (8.8%)
N1	665 (33.4%)
N2	791 (39.6%)
N3	362 (18.2%)
Stage	
I	42 (2.1%)
II	256 (12.8%)
III	859 (43.1%)
IVA	836 (42.0%)
Induction chemotherapy	
Yes	295 (14.8%)
No	1,698 (85.2%)
Concurrent chemotherapy	
Yes	1,276 (64.0%)
No	717 (36.0%)
Radiation dose to GTV	
Median (95% CI, Gy)	73.92 (69.96, 73.96)

Abbreviation: GTV: gross tumor volume; CI: confidence interval.

### 3.2 Conditional survival for entire study cohort

**FIGURE 1:**
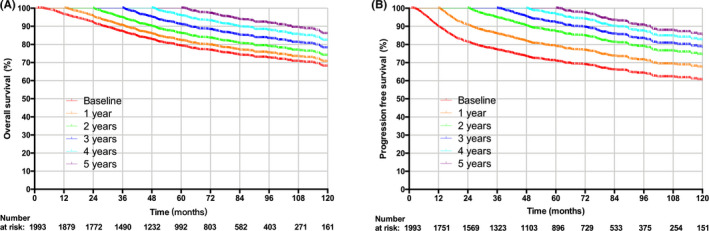
Conditional survival curves at baseline and 1 to 5 conditional years for the overall cohort: (A) conditional overall survival and (B) conditional progression‐free survival

Figure 1A demonstrates the OS curve estimated at initial diagnosis for entire cohort and the subsequent COS curves among patients who have survived for 1 to 5 years. The 5‐year COS probabilities were 79.5% (95% CI: 77.4% to 81.5%), 80.5% (95% CI: 78.2% to 82.6%), 83.2% (95% CI: 80.7% to 85.4%), 85.4% (95% CI: 82.5% to 87.8%), and 85.9% (95% CI: 82.1% to 88.9%), respectively, at conditional survival time of 1 to 5 years. Accordingly, the 5‐year CPFS were 76.6% (95% CI: 74.3% to 78.7%), 81.1% (95%CI: 78.6% to 83.3%), 83.2% (95%CI: 80.5% to 85.6%), 84.6% (95%CI: 81.5% to 87.2%), and 85.5% (95% CI: 81.9% to 88.5%) as estimated at 1 to 5 years of event‐free follow‐up (Figure 1B). Detailed depiction of the numbers and locations of censoring are provided in Figure S1.

### 3.3 Stage‐dependent adjusted conditional survival

**FIGURE 2:**
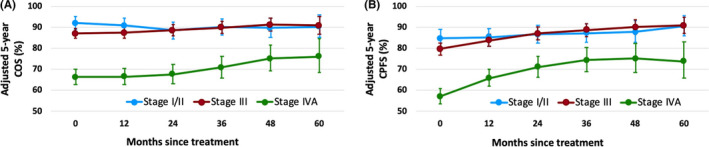
Adjusted 5‐year conditional survivals and corresponding 95% CI stratified by 8th AJCC stage adjusting for age and gender at baseline and 1 to 5 conditional years: (A) COS and (B) CPFS. Error bars indicate 95% confidence intervals. COS: conditional overall survival; CPFS: conditional progression‐free survival

Based on MCR models, AJCC stage, age at diagnosis and gender were independent predictors for risk of death and progression. To account for the confounding effect of covariates on survival function, stage specific conditional survivals were estimated controlling for age at diagnosis and gender. The estimated 5‐year COS and CPFS stratified by stage adjusting for age and gender are shown in Figure 2. Detailed survival data and corresponding CIs are provided in Table 2. For stage I/II, adjusted 5‐year COS maintained dramatically favorable rates at approximate 90% over the 5 years of follow‐up. Stage III demonstrated slightly lower 5‐year COS and CPFS than did stage I/II within the first 2 years and reached nearly equivalent probabilities since year 3 and thereafter. With regard to stage IVA, absolute increases of 5‐year probabilities of COS and CPFS were 9.8% and 16.8%, respectively after 5‐year event‐free follow‐up. Stage III and IVA shared similar dynamic pattern regarding both COS and CPFS. For COS, stage III and IVA both remained stable within the first 2 years and underwent appreciable increases thereafter. In terms of CPFS, stage III and IVA both exhibited remarkable elevations during the first 3 years and maintained a relatively steady level in years 4 and 5. The variations of survival probabilities over age and gender at each stage stratum are displayed in Figure S2 and S3. Younger age and female gender were correlated with more favorable COS and CPFS among all stage groups.

**TABLE 2:** Conditional 5‐year survival probabilities by stage adjusted for age and gender

	Baseline	1‐year	2‐year	3‐year	4‐year	5‐year
Stage I/II						
5‐y COS (%)	92.0	90.8	88.5	90.1	89.7	90.3
95% CI (%)	89.0, 95.1	87.5, 94.3	84.6, 92.6	86.3, 94.1	85.2, 94.5	85.2, 95.8
5‐y CPFS (%)	84.7	85.3	86.6	87.2	87.9	90.7
95% CI (%)	80.6, 88.9	81.2, 89.6	82.5, 91.0	82.9, 91.9	83.1, 93.1	86.1, 95.5
Stage III						
5‐y COS (%)	87.1	87.5	88.5	90.0	91.3	90.9
95% CI (%)	84.8, 89.6	85.0, 90.1	86.0, 91.2	87.2, 92.8	88.2, 94.4	86.9, 95.2
5‐y CPFS (%)	79.7	83.7	87.1	88.7	90.2	90.9
95% CI (%)	76.9, 82.6	80.9, 86.6	84.2, 90.1	85.7, 91.9	86.9, 93.6	87.2, 94.7
Stage IVA						
5‐y COS (%)	66.3	66.6	67.7	70.9	75.1	76.1
95% CI (%)	62.8, 70.0	62.8, 70.6	63.3, 72.3	65.9, 76.3	69.3, 81.4	68.5, 84.6
5‐y CPFS (%)	57.1	65.8	71.1	74.5	75.2	73.9
95% CI (%)	53.6, 60.8	61.9, 70.1	66.3, 76.2	69.1, 80.3	68.7, 82.2	65.7, 83.1

Abbreviation: COS: conditional overall survival; CPFS: conditional progression‐free survival; CI: confidence interval.

### 3.4 Stage‐dependent relative HRs predicting conditional OS and PFS

**FIGURE 3:**
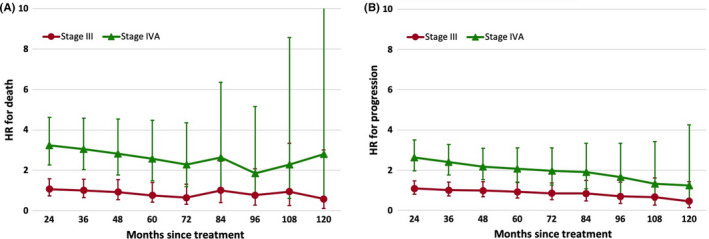
Multivariable Cox regression model‐based hazard ratios for (A) death and (B) progression according to AJCC 8th stage (referent: stage I/II; red line: stage III; green line: stage IVA). Error bars indicate 95% confidence intervals. HR: hazard ratio

Due to the PH assumption did not hold at baseline and the 1‐year landmark point in MCR analysis, we plotted the HR variations of AJCC stage for death and progression over time since 2‐year and onward (Figure 3). Relative to the referent stage I/II, stage III subgroup did not demonstrate significant higher HR in the context of death or progression during the entire follow‐up period. Stage IVA exhibited significantly higher risk for death (HR = 3.24, 95% CI: 2.26 to 4.63) and progression (HR = 2.63, 95% CI: 1.97 to 3.51) relative to the referent stage I/II at the second year after treatment. Thereafter, HR magnitude of death gradually declined to 2.63 (95% CI: 1.09, 6.36) and was no more significant from year 8 onward. With respect to progression, stage IVA manifested a constant and moderate decline of HR during the follow‐up, which was also no more significant since the conditional 8 years follow‐up.

### 3.5 Stage‐dependent annual hazard estimation of death and progression

**FIGURE 4:**
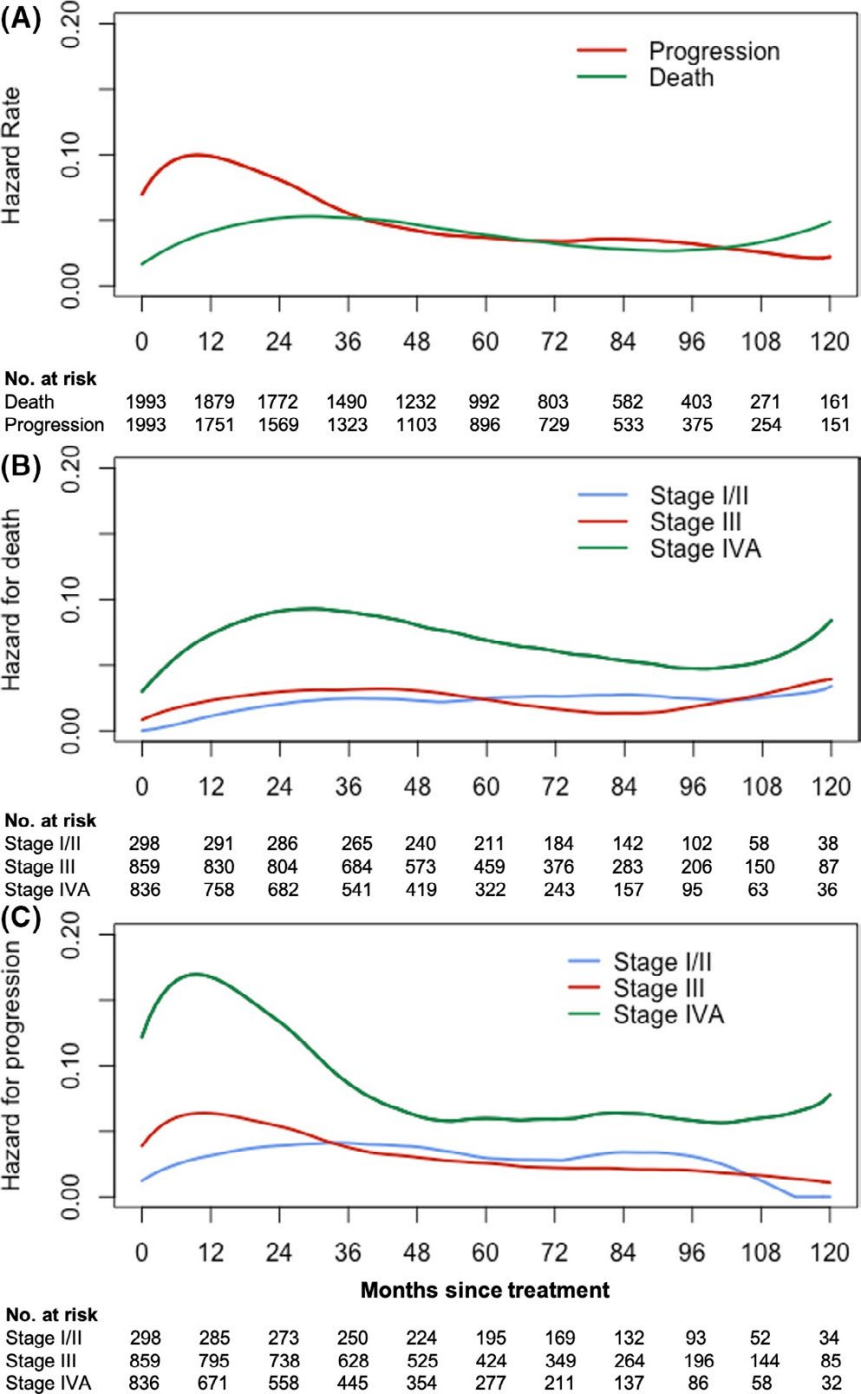
Failure hazards for entire cohort and respective 8th AJCC stages. (A) Smooth hazard rate for death (green line) and progression (red line) among entire study cohort; (B) Smooth hazard rates for death and (C) progression among patients with respective stages (Blue: stage I/II; red: stage III; green: stage IVA)

Annual hazard estimates of death and progression for entire cohort and respective AJCC stages are plotted in Figure 4. Detailed hazard data with 95% CIs are shown in Table S1.

Figure 4A displayed that annual hazard of death in entire cohort reached the peak to 5.5% (95% CI: 5.3% to 5.8%) between the 2nd and 4th year since treatment. Afterwards, the hazard gradually decreased to 2.7% (95% CI: 2.4% to 2.9%) till the 8th year and presented a moderate rebound between the 9th and 10th year of follow‐up. Accordingly, the progression hazard revealed a peak of 10.7% (95% CI: 10.3% to 11.2%) within the first 2 years and thereafter gradually decreased and maintained around 3% after 5 years of follow‐up and onward. Notably, the smooth curves of hazards for death and progression displayed an overlap since the conditional year 3 and onward.

Figure 4B plots the dynamic changes of death hazard over time for respective stages. Stage III shared similar and extremely low hazards of death with stage I/II, demonstrating less than 5% of hazard over a decade. Stage IVA presented much higher hazards of death over 10 years follow‐up, peaking to 9.6% (95% CI: 8.9% to 10.4%) at the interval of 2nd and 4th year. After maintaining decrease for additional 5 years, stage IVA presented a slight rebound between the 9th and 10th year after treatment.

Figure 4C depicts the annual progression hazard over time. Stage I/II cohort still demonstrated dramatically low hazards over a decade, with the maximum of 4.3% (95% CI: 3.7% to 4.8%) between the third‐ and fourth‐year follow‐up. Stage III and IVA subgroup both displayed the peak within the first 2 years of follow‐up, with the maximum hazard of 6.8% (95% CI: 6.3% to 7.3%) and 18.1% (95% CI: 16.9% to 19.4%), respectively. Beyond the 2nd year, progression risk of stage III gradually decreased to 1.8% (95% CI: 1.4% to 2.1%) till the 10th year. After passing through period of the peak hazard, stage IVA experienced a rapid decline during the following 3 years and then remained a relative stable rate of approximate 6% from year 5 onward.

## 4 DISCUSSION

To the best of our knowledge, this study provides the most comprehensive profile regarding how the prognosis evolves among non‐metastatic NPC patients receiving modern technique radiotherapy. On the basis of 1993 patients treated with IMRT, we obtained the dynamic estimates of OS and PFS at conditional surviving time and progression‐free time up to 10 years for the entire population as well as the subgroups stratified by the 8th AJCC stage. In addition, we further presented the estimates of dynamic absolute and relative hazard change for death and progression over a decade in the overall cohort and respective stage of patients. These findings highlight the importance of stage‐adapted treatment strategies and surveillance in patients with non‐metastatic NPC principally managed with IMRT.

There was only one additional study that we are aware of to investigate the conditional survivorship for NPC on the basis of SEER data from 1973 to 2007.^23^ This study demonstrated the respective 5‐year OS of 67% and 54% for local and regional diseases, which were apparently inferior to our baseline data. The advancement of radiation technique, adjunction of chemotherapy and ethnicity difference may contribute in part to the marked discrepancy between two studies. Moreover, due to the paucity of TNM stage and radiation technique in SEER database, the significance of this study in guiding current clinical practice was limited. In our study, we selected patients receiving mainstay technique radiotherapy and re‐staged them using 8th AJCC stage, enabling caregivers and patients to obtain the most updated prognostic information applicable to current clinical management.

Similar with the dynamic patterns of conditional survival in other tumors, COS and CPFS analysis verified the improved prognoses along with the elapse of event‐free follow‐up in non‐metastatic NPC. Different dynamic changes of conditional survivals were observed among various AJCC stages. Stage I/II maintained a persistent 5‐year COS of 90% and 6% of improvement in terms of 5‐year CPFS over the 10 years of follow‐up, reflecting a constantly favorable outcome in this early setting. Stage III demonstrated slightly lower 5‐year COS and CPFS than did stage I/II within the first 2 years and reached equivalent probabilities since year 3 and thereafter. This interesting finding raises a question as to whether de‐intensification of therapy for stage III is valid. Further prospective investigation of therapeutic outcomes among stage III patients is warranted. Stage IVA presented most impressive improvement in terms of both COS and CPFS whereas still drastically inferior to that of state I‐III across all conditional time points, justifying more intensive and prolonged management in this sub‐stage of patients. Besides, the persisting hazards of death and progression in stage IVA also accentuate the current incurability of this setting and further effort to improve the outcome is warranted.

According to popular guidelines in current practice, the follow‐up recommendations are generally uniform for patients harboring any stages of disease.^24^ However, it is apparent that substantial differences regarding conditional survival and failure hazard exist among sub‐stages. The “one fits all” follow‐up strategy is not appropriate anymore. Follow‐up intensity and frequency should be tailored at least according to TNM stage and ideally guided by a risk‐dependent manner incorporating multiple prognostic factors. Besides the description on trend of dynamic prognosis, our study also drew several hints that may shed light on the follow‐up schedule for NPC.

First, the annual hazard estimate of death and progression for overall population displayed an overlap from the third year and onward, implying that few patients would suffer additional disease progression after 3 years of follow‐up. These findings indicated that 3‐year follow‐up may be a valuable landmark and 3‐year PFS may be a valid endpoint for future clinical trial design in non‐metastatic NPC. In line with our results, previous SEER‐data based study also suggested 3 years as an appropriate follow‐up duration for trials including patients with localized NPC.^23^

Second, stage I/II cohort in our study maintained less than 5% of hazards of both death and progression over the 10 years of event‐free follow‐up. Furthermore, considering the existence of non‐cancer related death, the virtual death caused by cancer would have been even lower, warranting a lessening intensity and frequency of follow‐up schedule for this early setting.

Third, peak hazard of death and progression as well as unchanged 5‐year COS probabilities were consistently noted within the first 2 years of event‐free follow‐up for both stage III and IVA cohorts. Such trends have also been observed in other tumor types, such as melanoma and NK/T lymphoma.^9^, ^16^ On the basis of an endemic NPC cohort, a very recent publication reported that the peak of local and regional relapses occurred in the 18th to 24th months.^25^ Findings in the present study verified a similar peak period within 2 years post‐treatment in a non‐endemic cohort and provided additional evidence supporting the relatively closer follow‐up and more active adjuvant therapy within this 2‐year peak‐risk period. Furthermore, according to the absolute hazard estimation of each stage, a rigorous surveillance schedule for stage IVA and moderate follow‐up frequency for stage III within the first two years may be rational for NPC.

Fourth, after undergoing the peak period, hazard plots of stage III declined to 3.6% at the conditional 3rd year of follow‐up and maintained the decreasing manner thereafter, with comparable absolute hazard to that of stage I/II. This dynamic pattern implies that a similar follow‐up schedule to that of stage I/II is acceptable for stage III after 3 years of event‐free follow‐up. Likewise, stage IVA experienced rapid reduction of progression hazard after the peak period and subsequently entered the plateau of around 6% after 4 years of event‐free follow‐up. These dynamic trends suggest that 3‐year for stage III and 4‐year for stage IVA may be desirable landmarks for follow‐up strategy making.

Fifth, both death and progression HRs of stage IVA relative to stage I/II gradually diminished over period and the magnitude was no more significant since the 8th year after treatment, reflecting a lessening impact of stage assessed in decade bracket. Correspondingly, it may be rational for care providers to recommend an identical follow‐up schedule for NPC survivors after 8 years of event‐free follow‐up.

Based on the above clues, a stage‐adapted follow‐up diagram is proposed in Figure S4. Basically, a less frequent surveillance schedule (Level 3, e.g., annually) than current guideline may be adequate for stage I/II patients who harbor extremely and constantly low risk of hazard. Stage III is suggested to receive moderately frequent follow‐up (Level 2, e.g., every 6 months) for 3 years and then same schedule as stage I/II thereafter. Accordingly, stage IVA may require more intensive follow‐up during the first 4 years (Level 1, e.g., every 3 months) to timely detect disease progression and less intensive surveillance afterwards (Level 2) till the end of 8th year when progression risk comparable to that of stage I/II and III. Notably, an ideal clinical follow‐up care for cancer survivors should not only include surveillance for cancer recurrence or death, but also involve a broader spectrum, such as screening for second primary cancer, physical function assessment, long‐term psycho‐social effects of cancer and treatment and so forth.^26^ Therefore, we would like to highlight that all clues for the optimization of post‐therapeutic surveillance deduced from our study is based on considerations of cancer progression and death. More comprehensive data covering above‐mentioned aspects are needed to reach a more optimized surveillance plan.

Several limitations should be noted in our study. For example, due to patients in our database receiving treatment over a wide time span, emerging prognostic determinants such as Epstein–Barr Virus DNA (EBV‐DNA) data was not complete and thus not applicable for risk stratification. However, based on large real‐word cohort from non‐endemic areas, our findings and implications for clinical decision‐making are likely to have been more practical for non‐EBV‐related NPC patients. In addition, since there was a paucity of accurate patterns of failure or causes of death in a substantial proportion of patients, we did not make analysis on the detailed type of progression, cancer‐specific death or counterpart non‐cancer mortality in this study. Further research is warranted to identify the time cutoff when the death rate of early‐stage population approaches to that of background general population. Beyond this timepoint, the patients would not suffer with additional risk of dying from cancer and could be considered “cured of cancer.”

## 5 CONCLUSIONS

This study provides the most updated stage‐dependent overview of conditional outcomes as well as dynamic failure hazards for non‐metastatic NPC in the era of IMRT and AJCC 8th stage. Several stage specific landmark timepoints are noticeable and the reduction of surveillance frequency may be justified for patients outliving the landmark time. These findings provide important implications for dynamic and personalized decision‐making in terms of both clinical management and surveillance counseling.

## CONFLICT OF INTEREST

C.H. has received grants from the National Cancer Institute and RTOG Foundation, and personal fees from Merck & Co. outside the submitted work. Other authors declare no conflict of interest.

## AUTHOR CONTRIBUTIONS

Jingbo Wang: Conceptualization, data curation, formal analysis, investigation, methodology, writing‐original draft and editing. Xiaodong Huang: Conceptualization, data curation, formal analysis, investigation, writing‐review and editing. Shiran Sun: Conceptualization, data curation, writing‐review and editing. Kai Wang: Conceptualization, writing‐review and editing. Yuan Qu: Conceptualization, writing‐review and editing. Xuesong Chen: Conceptualization, writing‐review and editing. Runye Wu: Conceptualization, writing‐review and editing. Ye Zhang: Conceptualization, writing‐review and editing. Qingfeng Liu: Conceptualization, writing‐review and editing. Jianghu Zhang: Conceptualization, writing‐review and editing. Jingwei Luo: Conceptualization, writing‐review and editing. Jianping Xiao: Conceptualization, writing‐review and editing. Li Gao: Conceptualization, writing‐review and editing. Guozhen Xu: Conceptualization, writing‐review and editing. Chen Hu: Methodology, writing‐review and editing. Yexiong Li: Conceptualization, writing‐review and editing. Junlin Yi: Conceptualization, formal analysis, funding acquisition, investigation, project administration, resources, supervision, writing‐original draft, and writing‐review and editing.

## ETHICAL APPROVAL

This study was performed in accordance with the Declaration of Helsinki and approved by the institutional review board of National Cancer Center (No. NCC2462).

## Supporting information

Fig S1

Fig S2

Fig S3

Fig S4

Table S1

## Data Availability

The data that support the findings of this study are available from the corresponding author upon reasonable request.
