# Supplementary figures and images for: Stage‐dependent conditional survival and failure hazard of non‐metastatic nasopharyngeal carcinoma after intensity‐modulated radiation therapy: Clinical implications for treatment strategies and surveillance
Source: Cancer Med. 2021 May 6;10(11):3613–21. doi: 10.1002/cam4.3917 (PMC8178506; doi:10.1002/cam4.3917)

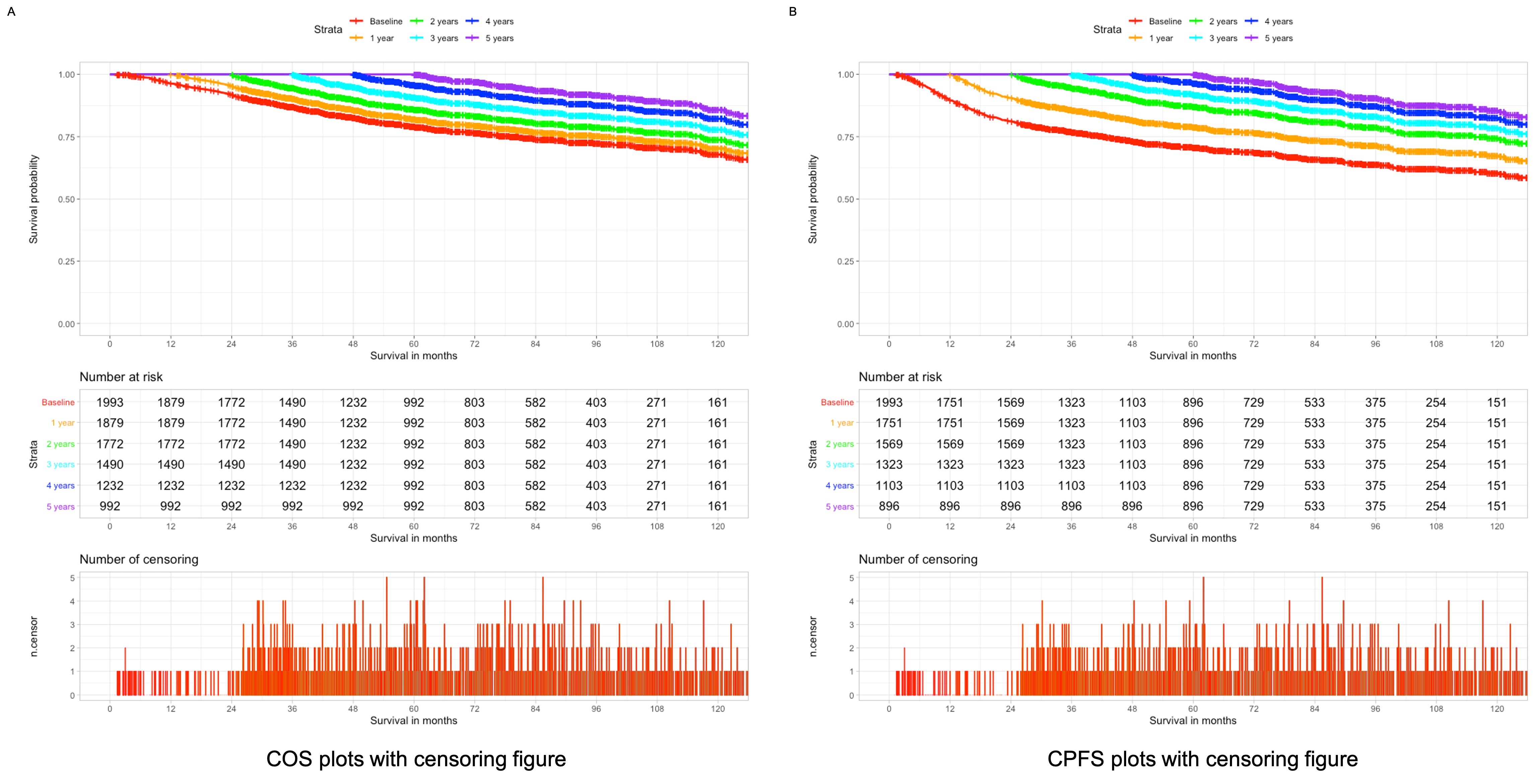

Supplement: Supplementary file 1 — Fig S1 [file CAM4-10-3613-s004.tif]

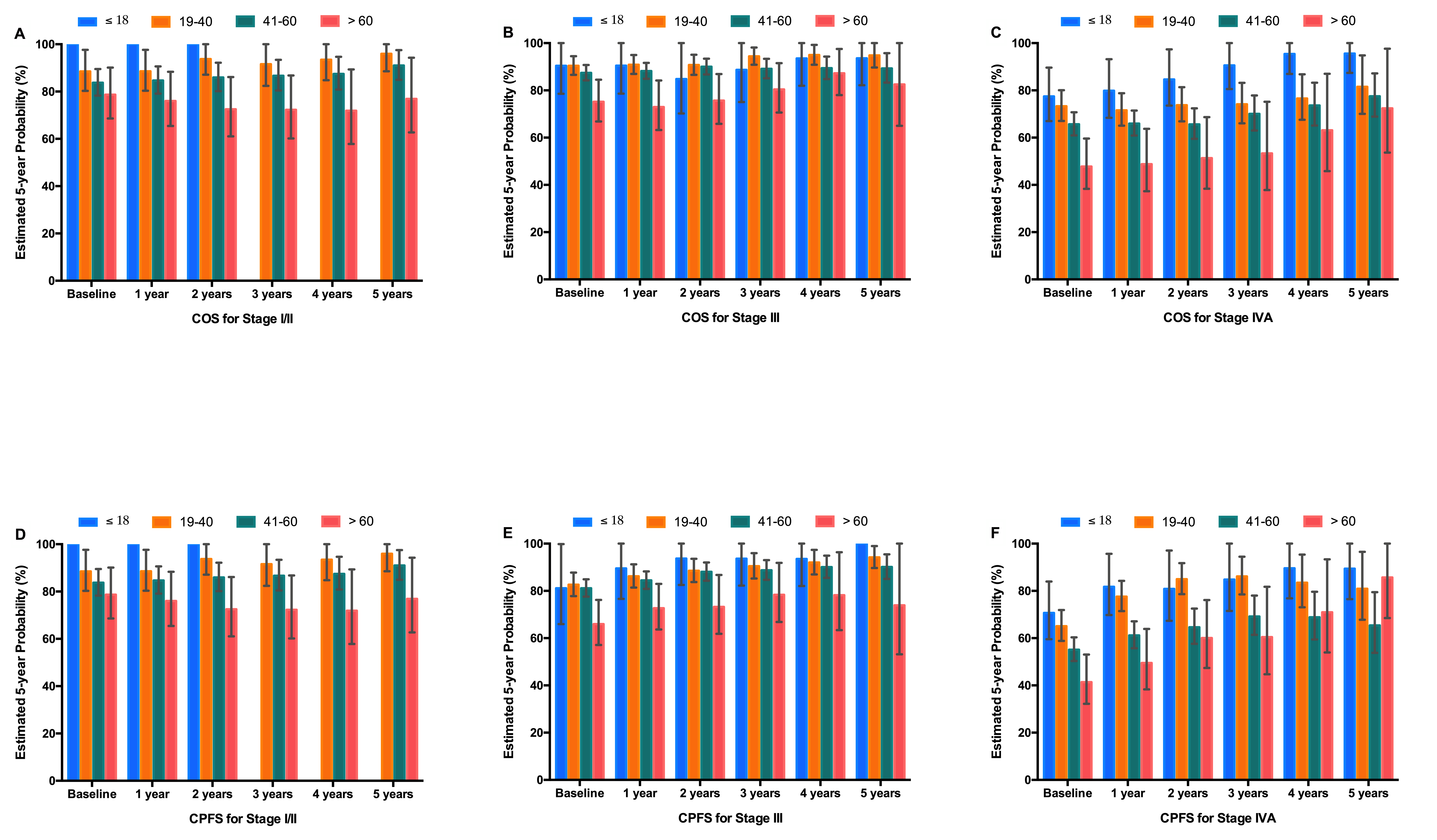

Supplement: Supplementary file 2 — Fig S2 [file CAM4-10-3613-s001.tiff]

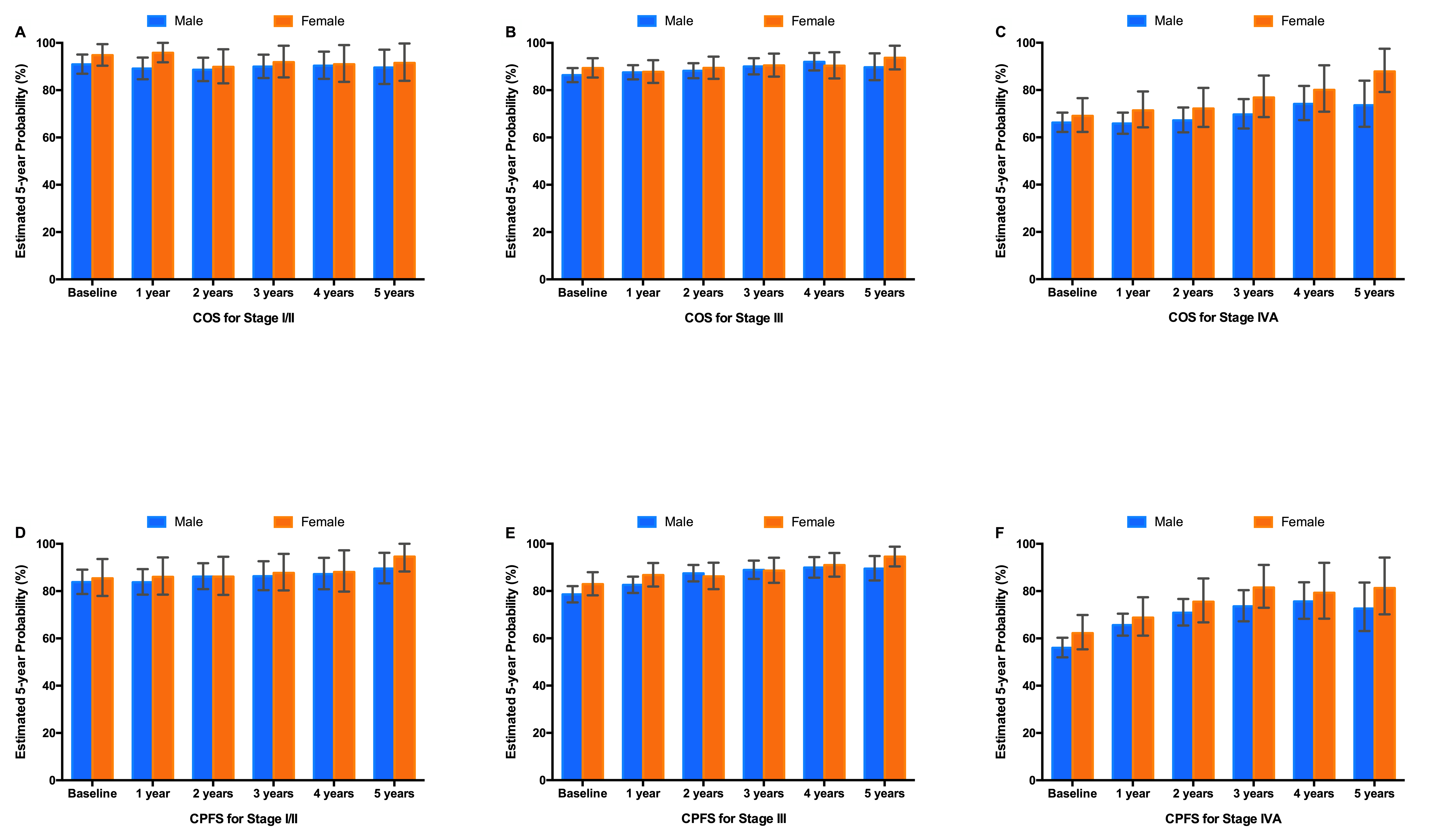

Supplement: Supplementary file 3 — Fig S3 [file CAM4-10-3613-s003.tiff]

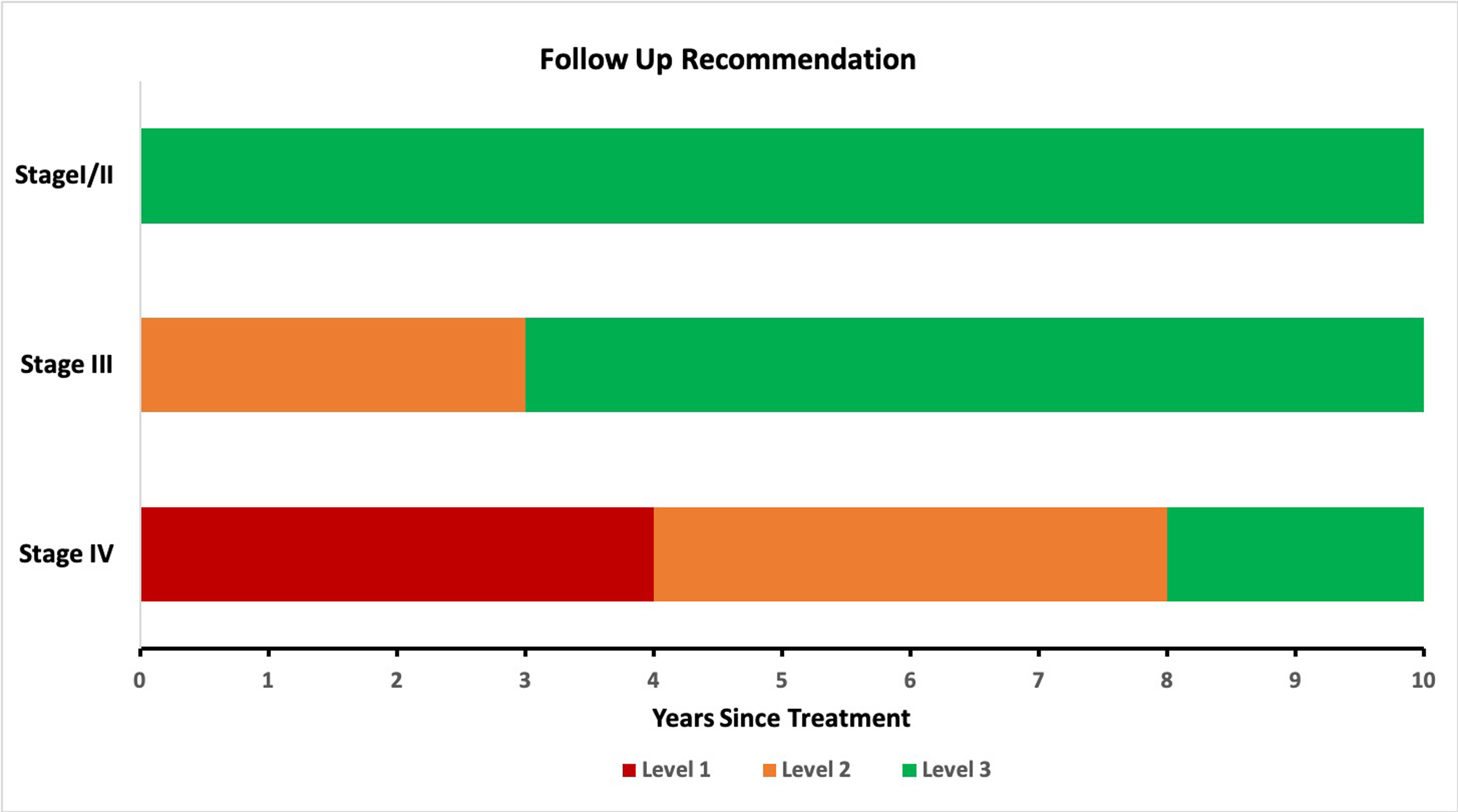

Supplement: Supplementary file 4 — Fig S4 [file CAM4-10-3613-s002.tif]
