# Supplementary material for: Stage‐dependent conditional survival and failure hazard of non‐metastatic nasopharyngeal carcinoma after intensity‐modulated radiation therapy: Clinical implications for treatment strategies and surveillance
Source: Cancer Med. 2021 May 6;10(11):3613–21. doi: 10.1002/cam4.3917 (PMC8178506; doi:10.1002/cam4.3917)
Supplement: Supplementary file 5 — Table S1 [file CAM4-10-3613-s005.docx]

**Supplementary Materials**

**Supplementary Table 1.** Annual hazard of death and progression for entire population and respective AJCC 8th substages.

**Supplementary Figure 1.** Conditional survival curves at baseline and 1 to 5 conditional years for the overall cohort with censoring figures: (A) conditional overall survival and (B) conditional progression-free survival. COS: conditional overall survival; CPFS: conditional progression-free survival.

**Supplementary Figure 2.** The variations of survival probabilities over age at each stage stratum. COS: conditional overall survival; CPFS: conditional progression-free survival.

**Supplementary Figure 3.** The variations of survival probabilities over gender at each stage stratum. COS: conditional overall survival; CPFS: conditional progression-free survival.

**Supplementary Figure 4.** Follow-up recommendation based on current study for non-metastatic NPC: Level 1 indicates the most frequent follow-up schedule; level 2 indicates the intermediately frequent surveillance and level 3 represents the least frequent follow-up.

| **Supplementary Table 1. Annual hazard of death and progression for entire population and respective AJCC 8th substages** | | | | | | | | | | | | |
| --- | --- | --- | --- | --- | --- | --- | --- | --- | --- | --- | --- | --- |
|  | 1-year | 2-year | 3-year | 4-year | 5-year | 6-year | 7-year | 8-year | 9-year | 10-year |  |  |
| Entire cohort | | | | | | | | | | | |  |
| Annual hazard of death (%) | 3.7 | 4.7 | 5.5 | 5.3 | 4.4 | 3.2 | 3.4 | 2.2 | 2.7 | 3.7 |  |  |
| 95% CI (%) | 3.5, 3.9 | 4.4, 4.9 | 5.3, 5.8 | 5.0, 5.6 | 4.2, 4.7 | 3.0, 3.4 | 3.1, 3.6 | 2.0, 2.4 | 2.4, 2.9 | 3.2, 4.2 |  |  |
| Annual hazard of progression (%) | 10.7 | 10.0 | 5.5 | 4.9 | 3.6 | 3.0 | 4.2 | 2.9 | 3.5 | 2.5 |  |  |
| 95% CI (%) | 10.3, 11.2 | 9.5, 10.4 | 5.2, 5.8 | 4.6, 5.1 | 3.4, 3.9 | 2.8, 3.2 | 3.9, 4.5 | 2.6, 3.1 | 3.1, 3.9 | 2.1, 2.8 |  |  |
| Stage I/II | | | | | | | | | | | |  |
| Annual hazard of death (%) | 0.7 | 1.4 | 2.5 | 2.8 | 2.3 | 2.1 | 4.3 | 0.8 | 3.7 | 2.1 |  |  |
| 95% CI (%) | 0.6, 0.8 | 1.2, 1.5 | 2.2, 2.8 | 2.4, 3.1 | 2.0, 2.6 | 1.8, 2.3 | 3.6, 4.9 | 0.6, 1.0 | 2.9, 4.5 | 1.4, 2.6 |  |  |
| Annual hazard of progression (%) | 2.7 | 3.9 | 3.4 | 4.3 | 3.9 | 2.8 | 2.6 | 3.5 | 4.1 | 0 |  |  |
| 95% CI (%) | 2.4, 3.1 | 3.4, 4.3 | 3.1, 3.8 | 3.7, 4.8 | 3.3, 4.5 | 2.4, 3.2 | 2.2, 3.0 | 2.9, 4.2 | 3.2, 5.0 | NA |  |  |
| Stage III | | | | | | | | | | | |  |
| Annual hazard of death (%) | 2.5 | 2.2 | 3.3 | 3.2 | 3.1 | 2.0 | 0.9 | 1.6 | 1.7 | 3.4 |  |  |
| 95% CI (%) | 2.3, 2.6 | 2.0, 2.4 | 3.1, 3.6 | 2.9, 3.4 | 2.8, 3.4 | 1.8, 2.2 | 0.8, 1.0 | 1.4, 1.8 | 1.4, 1.9 | 2.8, 4.0 |  |  |
| Annual hazard of progression (%) | 6.7 | 6.8 | 3.6 | 3.4 | 2.5 | 2.1 | 2.6 | 1.7 | 1.8 | 1.8 |  |  |
| 95% CI (%) | 6.3, 7.2 | 6.3, 7.3 | 3.4, 3.9 | 3.2, 3.7 | 2.3, 2.8 | 1.9, 2.3 | 2.3, 2.9 | 1.5, 2.0 | 1.5, 2.0 | 1.4, 2.1 |  |  |
| Stage IVA | | | | | | | | | | | |  |
| Annual hazard of death (%) | 6.1 | 8.8 | 9.6 | 9.5 | 7.5 | 5.7 | 6.8 | 4.8 | 3.8 | 6.1 |  |  |
| 95% CI (%) | 5.7, 6.5 | 8.1, 9.4 | 8.9, 10.4 | 8.6, 10.3 | 6.7, 8.2 | 5.1, 6.4 | 5.8, 7.7 | 4.0, 5.7 | 3.0, 4.7 | 4.4, 7.8 |  |  |
| Annual hazard of progression (%) | 18.1 | 16.7 | 9.2 | 7.3 | 5.0 | 4.5 | 8.5 | 4.6 | 7.0 | 6.6 |  |  |
| 95% CI (%) | 16.9, 19.4 | 15.4, 18.0 | 8.3, 10.0 | 6.5, 8.0 | 4.5, 5.6 | 4.0, 5.1 | 7.2, 9.7 | 3.7, 5.4 | 5.4, 8.7 | 4.7, 8.6 |  |  |
| CI: confidence interval. | | | | | | | | | | | |  |
